# Akephaloid Monster

**Published:** 1870-05

**Authors:** N. B. Larsh

**Affiliations:** Nebraska City, Neb.


Article VI.—*Akephaloid Monster.* By N. B. Larsh, M.D., Nebraska City, Neb.

Accompanying this communication, I send you a photograph of an akephaloid monster, resembling in many respects the case recently reported to your journal by Dr. E. R. Hutchins, of Philadelphia.*

Mrs. H., aet. 32, the mother of four children, all healthy and well developed, was confined at full time, January 16th, last. About four weeks previous to this date she was surprised by the sudden and copious discharge of a large quantity of water, and supposing that labor had commenced or was about to commence, sent for her physician. No further symptoms of labor occurred until the date above named. Dr. Congar, who was called to attend the case, recognized some abnormal deviation in the presenting part, and the labor being tedious, sent for me to assist him.

Before my arrival, however, the mother had given birth to a still-born fœtus, such as you see delineated in the photograph. There was nothing peculiar or unusual in the labor, except, perhaps, an unusually large quantity of liquor amnii. No decomposition had taken place. The fœtus was about the average weight, with limbs well formed.

The head, however, presented the most remarkable deformity. The face and anterior part of the cranium, looking upward, were placed upon the shoulders. The mastoid processes spreading out, were in coaptation with the spine of the scapula on both sides.

From about the fourth dorsal vertabræ there extended upwards a livid surface of denuded, or what appeared to be denuded, muscular fibre, which terminated in a large livid symmetrical tumor, projecting from the cranium. The spinous processes of the vertabræ above the fourth dorsal, were wholly wanting, as was also the bony wall of the cranium from whence the tumor projected.

The external ears projected forward and opened anteriorly, being mere folds of the skin, without the usual helices.

As no dissection of the fœtus was made, the anatomical description is necessarily imperfect.

* The general appearance of this monstrosity is so similar to Dr. Hutchins' case, (*Journal*, Dec. 1869, p. 704,) that the wood cut is thought unnecessary. —Ed.

